# Mechanical Research on Reinforced Concrete Materials

**DOI:** 10.3390/ma16216892

**Published:** 2023-10-27

**Authors:** Wei Wang

**Affiliations:** Key Laboratory of Impact and Safety Engineering, Ningbo University, Ministry of Education, Ningbo 315211, China; wangwei7@nbu.edu.cn

Reinforced concrete (RC) is a commonly used construction material in civilian and military buildings due to its superior material characteristics compared to steel and timber (e.g., higher durability, corrosion resistance, and fire resistance). These inherent properties of reinforced concrete make it suitable for the construction of most civil engineering structures, for example, bridges, dams, nuclear containment structures, protective/defense structures, and residential/embassy buildings. Concrete is a frequently used material that is subjected to intense dynamic loading in civil and defense engineering, such as blast and impact loadings, which can induce high pressure, high strain rates, and a large amount of strain in concrete structures. The response of such structures is very complex due to the effects of high inertia, high strain rate, high temperature, and shock waves traveling through the reinforced concrete. Although the mechanical behavior of reinforced concrete has been investigated by many researchers using experimental and theoretical approaches for 200 years, establishing an accurate and comprehensive description of the actual mechanical behavior exhibited by reinforced concrete under service and ultimate conditions remains a challenge.

The purpose of this Special Issue is to investigate theoretical, experimental, and numerical studies on the mechanical properties of reinforced concrete materials, to evaluate the general deformation response, damage evolution, and failure patterns of ordinary and high-performance reinforced concrete materials under various loading conditions (e.g., quasi-static, dynamic, fatigue, and impact).

Dong et al. [1] studied the influence of relative density and water content on the dynamic characteristics of coral sand using an SHPB device, and obtained a stress–strain curve of the material under uniaxial strain compression with different relative densities and water contents. The results showed that the strain rate became less sensitive to the stiffness of coral sand with an increase in relative density.

Wu et al. [2]. studied the effects of different reinforcement distributions and blasting distances on the antiknock performance of RC plates. The results showed that the degree of damage on a single-layer steel plate was more serious than that on a double-layer steel plate under contact explosion and non-contact explosion. When the scale distance was the same, the degree of damage on single-layer and double-layer steel bars first increased, and then, decreased with an increase in distance.

Ma et al. [3] studied the structural behavior of full-iron tailing-reinforced concrete (FITRC) columns under high eccentric loading. Six FITRC column and two CRC column specimens were examined to investigate the effects of different raw materials, section dimensions, and eccentricities on the mechanical behavior of RC columns under high eccentric compression. They found that under high eccentric short-term loads, the failure modes of the FITRC and CRC columns were similar, and their failure manifested as yielding of the tensile and compressive rebars and concrete crushing in the compression zone. Additionally, the moment augmentation factor of the FITRC columns should be 1.15 for safety reasons.

Zhang et al. [4] investigated the bond behavior between PVA-fiber-reinforced concrete and steel rebar corroded in a chloride environment. The effects of PVA fibers and corrosion loss on bond behavior were clarified. They found that with the increase in the fibers, the corrosive cracking became more obvious. Moreover, the PVA fiber generally showed a negative effect on bond behavior but a positive effect on the descending branches in the case of splitting failure.

In their innovative study, Wang et al. [5] used superfine cement to modify coral aggregates. The effects of the water–cement ratio and curing time on the water absorption and strength of modified coral aggregates were investigated. The experimental results showed that when the water–cement ratio exceeded 1.25, the slurry did not form a shell on the surface of the coral aggregates and the water absorption of the coral aggregates increased significantly. The strength of the modified coral aggregates cured for a short duration was slightly lower than that of unmodified coral aggregates, while the strength of the coral aggregates cured for 28 days was approximately 20% higher than that of the unmodified coral aggregates.

Wang et al. [6] analyzed the anti-explosion performance of RC slabs by studying the residual bearing capacity of RCs slab under close-in blast loading, and it was found that the load distribution of the RC slabs was extremely uneven under these conditions. The range and degree of damage of the low-reinforcement-ratio slab were significantly higher than those of the high-reinforcement-ratio slab. Increasing the reinforcement ratio can inhibit the crack extension and reduce the residual displacement of a component, and reduce the decrease in bearing capacity after damage.

Lai et al. [7] presented a new method to study the dynamic mechanical properties of concrete under low pressure and a high strain rate via the inversion of spherical wave propagation. The dynamic parameters of the rate-dependent constitutive relation of elastic concrete were determined by measuring the velocity histories of spherical waves. The numerical constitutive relation was expressed in the form of distortion, and it was found that the distortion law had an obvious rate effect. The research results showed that the strain rate of concrete had a considerable effect when the strain rate of concrete was in the range of 10^2^ s^−1^.

Zhang et al. [8] used a true triaxial split–Hopkinson pressure bar system to conduct dynamic compression tests on ultra-high-performance concrete with different steel fiber contents (0.5%, 1% and 2%) under triaxial constraint. The results showed that the dynamic peak axial stress–strain and dynamic peak lateral stress–strain of UHPC are very sensitive to the strain rate, and the dynamic strength failure criterion of UHPC under triaxial constraints was established.

Xiong et al. [9] proposed a non-explosive method to simulate the explosion load of reinforced concrete slabs, and conducted experiments and numerical simulations to evaluate the effectiveness of this method. The experimental results showed that there was almost no difference between the pressure wave and the pressure peak produced by the simulated explosion load and the actual explosion. In addition, cone rubber was more suitable than plane rubber as an impact buffer material when simulating the explosion load.

Wu et al. [2] carried out field chemical explosion experiments on RC slabs with the same reinforcement ratio but different reinforcement distributions, and with the same blast distance but different scale distances. The effects of different reinforcement distributions and explosion distances on the antiknock performance and damage forms of RC plates were studied. It was found that double-layer and multi-layer reinforcement improved the blasting resistance of RC plates.

Yang et al. [10] studied shear horizontal (SH) wave scattering using a circular pipeline in an inhomogeneous concrete with density variation. A model of inhomogeneous concrete with density variation was established in the form of a polynomial–exponential coupling function. The results showed that the inhomogeneous density parameters, the wave number of the incident wave, and the angle of the incident wave in concrete were important factors affecting the distribution of dynamic stress around the circular pipe in concrete with inhomogeneous density.

Dong et al. [11] established theoretical models to calculate the mass erosion and heat conduction of projectile noses, including models of cutting, melting, the heat conduction of flash, and the conversion of plastic work into heat. The coupling numerical calculation of the erosion and heat conduction of the projectile nose showed that melting erosion was the main factor affecting mass loss at high-speed penetration, and the mass erosion ratio of melting and cutting was related to the initial velocity.

Wang et al. [12] used SHPB to study the dynamic mechanical properties of UR50 ultra-early-strength cement-based self-compacting high-strength material. The experimental results showed that the dynamic compressive strength increased with an increase in strain rate, which had an obvious strain-rate-strengthening effect. The damage variables at different strain rates were fitted, and it was found that an increase in strain rate had an obstructive effect on the increase in the damage variable and the increase rate.

The research papers included in this Special Issue demonstrate the usefulness of global institutional collaborations in helping to share knowledge and deepen our understanding of various topics in the field of reinforced concrete materials.

Finally, the editors would like to express their sincere thanks to the authors who actively participated in the peer review process, and to the editorial team of *Materials* for their cooperation and support.
